# Role of Osteocalcin in the Regulation of Glucose Metabolism in Children and Adolescents with Obesity

**DOI:** 10.3390/nu18203418

**Published:** 2026-10-18

**Authors:** Serena Sbilordo, Selene Francesca Anna Drago, Antonino Catalano, Giuseppina Zirilli, Giuseppina Salzano, Fortunato Lombardo, Stefano Passanisi, Bruno Bombaci, Loredana Grasso, M’Hammed Aguennouz, Angela Alibrandi, Vincenzo Macaione, Mariella Valenzise

**Affiliations:** 1 Department of Human Pathology of Adulthood and Childhood, University of Messina, 98125 Messina, Italy; serenasbilordo@hotmail.it (S.S.); giuseppina.zirilli@polime.it (G.Z.); giuseppina.salzano@unime.it (G.S.); fortunato.lombardo@unime.it (F.L.); stefano.passanisi@unime.it (S.P.); bruno.bombaci@studenti.unime.it (B.B.); angela.alibrandi@unime.it (A.A.); 2 Department of Chemical, Biological, Pharmaceutical and Environmental Sciences, University of Messina, 98166 Messina, Italy; sedrago@unime.it; 3 Department of Clinical and Experimental Medicine, University of Messina, 98125 Messina, Italy; catalano.antonino@unime.it (A.C.); loredana.grasso@unime.it (L.G.); aguenoz.mhommed@unime.it (M.A.); vincenzo.macaione@unime.it (V.M.); 4 Centro di Riferimento Regionale Obesità Infantile, AOU G. Martino-Messina, 98125 Messina, Italy

**Keywords:** osteocalcin, pediatric obesity, insulin resistance, HOMA-IR, glucose metabolism

## Abstract

Background: Osteocalcin (OC) is a bone-derived hormone increasingly implicated in glucose and lipid metabolism. Although its metabolic role has been extensively investigated in adults, evidence in pediatric populations remains limited. This exploratory study aimed to evaluate the relationship between serum OC levels and glucose metabolism in children and adolescents with obesity. Methods: A single-center, retrospective study was conducted in a cohort of pediatric patients with essential obesity. Auxological and biochemical parameters were assessed, including fasting glucose, insulin, lipid profile, liver enzymes, thyroid function, calcium–phosphorus metabolism, vitamin D_3_, parathormone (PTH), *C*-terminal telopeptide of type I collagen (CTX), and OC. Insulin resistance was evaluated using the HOMA-IR index (>2.5 considered positive). Correlation analyses were performed to assess the associations between serum OC levels and metabolic, biochemical, and clinical parameters. Results: Twenty-two of 33 patients (66.7%) had insulin resistance (HOMA-IR > 2.5) despite preserved glucose tolerance. Mean fasting glucose was 88.1 ± 10.83 mg/dL, fasting insulin 24.9 ± 13 μU/mL, and mean serum OC 112.1 ± 56.1 ng/mL. Serum OC levels were positively correlated with fasting insulin (r = 0.41, *p* = 0.023) and HOMA-IR (r = 0.46, *p* = 0.013). Conclusions: In children with obesity and preserved glucose tolerance, higher serum OC levels were associated with insulin resistance. Further studies in larger pediatric cohorts are needed to clarify this association and evaluate the potential role of OC as a biomarker of early metabolic dysregulation.

## 1. Introduction

Childhood obesity represents one of the most critical public health challenges worldwide. According to recent estimates from the World Health Organization (WHO) [1] and large-scale epidemiological studies, the prevalence of overweight and obesity among children and adolescents has risen dramatically over the past four decades. This trend has reached pandemic proportions and persists in low- and middle-income countries [2,3]. Pediatric obesity is associated with early-onset metabolic derangements, including insulin resistance (IR), dyslipidemia, low-grade chronic inflammation, increased risk of progression to type 2 diabetes mellitus (T2DM) and cardiovascular disease in adulthood [4,5,6].

IR in childhood is characterized by reduced insulin-mediated glucose uptake, primarily in skeletal muscle, and by impaired suppression of hepatic glucose production [7]. IR plays a key role in the development of metabolic syndrome and is associated with an increased risk of future cardiometabolic complications [8]. Early detection of insulin resistance is therefore essential, and several surrogate markers are commonly used in pediatric populations. Among these, the Homeostasis Model Assessment of Insulin Resistance (HOMA-IR) is widely accepted as a practical tool for assessing insulin resistance [9]. HOMA-IR values above 2.5 are generally considered indicative of insulin resistance, although cut-offs may vary according to age, sex, and pubertal stage [10,11]. Moreover, IR is closely associated with dysregulated adipokine secretion, including reduced adiponectin levels, as well as with alterations in bone-derived endocrine signaling. In particular, undercarboxylated osteocalcin (ucOC) has been shown to influence both insulin secretion and insulin sensitivity, highlighting the emerging role of the skeleton in glucose homeostasis [12,13].

In addition to its well-established metabolic consequences, childhood obesity profoundly affects skeletal development and has an impact on bone metabolism. Excess adiposity during growth may alter the complex physiological interactions between bone, adipose tissue, and energy metabolism, potentially influencing peak bone mass acquisition and long-term skeletal health [14,15]. Increasing evidence has established bone as an active endocrine organ and key modulator of metabolism [16]. Through the secretion of bone-derived hormones, notably osteocalcin (OC), which plays a crucial role in bone-metabolic crosstalk, the skeleton contributes to the regulation of glucose and lipid homeostasis [17,18].

OC, a 49–amino acid protein secreted by osteoblasts, is the most prevalent non-collagenous component of the unmineralized bone matrix and represents about 15% of its total protein content. OC exists in two forms: carboxylated (cOC) and undercarboxylated (ucOC), of which only ucOC exerts endocrine functions [13]. ucOC acts hormonally on multiple tissues, including pancreatic islets, adipose tissue, liver, skeletal muscle, and intestine, and its biological effects are mediated via the G protein–coupled receptor family C group 6 member A (GPRC6A) [19,20].

The interaction between OC and GPRC6A activates the IP_3_–Ca^2+^ signaling cascade through phospholipase C (PLC), stimulating the secretion of insulin and adiponectin [21]. Additionally, the adenylyl cyclase–cAMP–PKA pathway triggers MEK–ERK signaling, promoting β-cell proliferation, adipocyte differentiation, and enhanced insulin sensitivity [22,23].

Recent studies have explored the effects of ucOC on the expression of adipocyte adhesion molecule (ACAM) [24]. ACAM is a cell–cell adhesion protein expressed in adipocytes. Furthermore, ucOC may modulate integrins, a superfamily of cell-adhesion receptors involved in interactions with extracellular matrix ligands. Emerging evidence suggests that OC modulates glucose and lipid metabolism by regulating the expression of ACAM, integrin αVβ3, GLUT1, and GLUT8, directly influencing adipocyte functions. Moreover, the extracellular matrix of adipose tissue has been shown to regulate adipocyte activity and insulin sensitivity under pathophysiological conditions [24,25].

OC has been reported to improve insulin sensitivity through multiple mechanisms, including indirect effects mediated by adiponectin secretion from adipose tissue. In skeletal muscle, activation of GPRC6A promotes glucose and fatty acid uptake and enhances their utilization, particularly during fasting and exercise [26]. It also stimulates the release of cytokines, most notably interleukin-6 (IL-6), which contributes to the regulation of hepatic gluconeogenesis and promotes fatty acid mobilization from adipose tissue [12,27]. Several cytokines play key roles in metabolic homeostasis by regulating glucose metabolism, hepatic gluconeogenesis, and fatty acid catabolism [28]. The interaction of adiponectin with its receptors, AdipoR1 and AdipoR2, mediates tissue-specific metabolism effects. In skeletal muscle, AdipoR1 activation promotes glucose and fatty acid uptake, whereas in the liver, AdipoR2 suppresses the expression of gluconeogenic enzymes, reduces hepatic glucose output, and enhances insulin sensitivity [29,30].

Collectively, these mechanisms suggest that OC may contribute to the complex crosstalk between bone, pancreatic, adipose, and muscle tissues, linking skeletal remodeling to systemic glucose metabolism and insulin sensitivity. Therefore, the present study aimed to evaluate the relationship between bone remodeling biomarkers, particularly OC, and glucose metabolism in children and adolescents with essential obesity.

## 2. Materials and Methods

A single-center, retrospective study was conducted in a cohort of pediatric patients with essential obesity followed at the Regional Reference Center for Pediatric Obesity, Pediatric Unit, A.O.U. G. Martino, Messina, Italy. Eligible patients were identified; clinical, auxological, and biochemical data were retrospectively collected from medical records. All data were fully anonymized and de-identified prior to analysis. Given the retrospective observational design and the use of exclusively anonymized data routinely collected as part of standard clinical care, approval by the Ethics Committee was not required.

The study population included 33 children and adolescents with obesity (13 males and 20 females), with a mean age of 10.37 ± 2.9 SD years and a body mass index (BMI) at or above the 95th percentile for age and sex according to pediatric reference standards. The diagnosis of obesity was established based on a detailed medical history, a comprehensive physical examination, and extensive auxological and laboratory evaluations.

The following auxological and biochemical parameters were collected for each patient:

- Auxological assessment: bone age, body weight, height, and BMI.
- Biochemical parameters: fasting plasma glucose and insulin levels, total cholesterol, high-density lipoprotein (HDL) cholesterol, low-density lipoprotein (LDL) cholesterol, triglycerides, liver transaminases (aspartate aminotransferase [AST/GOT] and alanine aminotransferase [ALT/GPT]), uric acid, thyroid-stimulating hormone (TSH), free triiodothyronine (FT3), free thyroxine (FT4), insulin-like growth factor 1 (IGF-1), calcium, phosphorus, alkaline phosphatase, 25-hydroxyvitamin D, parathyroid hormone, *C*-terminal telopeptide of type I collagen (CTX), and OC.
- Assessment of insulin resistance: estimated using the homeostatic model assessment of insulin resistance (HOMA-IR), calculated according to the following formula: HOMA-IR = [fasting plasma glucose (mmol/L) × fasting insulin (mU/L)]/22.5. Fasting plasma glucose concentration was converted from mg/dL to mmol/L, when necessary, before calculating the HOMA-IR index. A value above 2.5 was considered indicative of insulin resistance [31].

A subgroup analysis was performed in participants with insulin resistance (HOMA-IR > 2.5) to characterize glucose and insulin profiles, including fasting HOMA-IR and serum insulin and glucose concentrations measured at baseline and at 30, 60, 90, and 120 min during the oral glucose tolerance test (OGTT).

### Statistical Analysis

Descriptive statistics were performed to characterize the study population. Categorical variables were reported as absolute frequencies and percentages, whereas continuous variables were expressed as mean ± standard deviation (SD). The association between serum osteocalcin (OC) and clinical, auxological, and biochemical variables was assessed using Spearman’s rank correlation coefficient (ρ), selected according to the non-parametric distribution of the variables. Correlation analyses were performed to investigate the potential relationships between circulating OC concentrations and markers of metabolic status, insulin resistance, lipid metabolism, liver function, calcium–phosphorus metabolism, and other relevant clinical and biochemical variables. Given the exploratory nature of the study and the limited sample size, these analyses were considered hypothesis-generating. A pre-set significance level of 0.050 was adopted, with *p*-values < 0.050 considered statistically significant. Statistical analysis was conducted using MedCalc version 10.3 (MedCalc Software bvba, Ostend, Belgium).

## 3. Results

The 33 enrolled patients ranged in age from 5.3 to 15.5 years (exact age calculated from birthdate to hospital admission), reflecting the broad pediatric age range routinely evaluated at our center. At diagnosis, patients had a mean BMI of 32.01 ± 5.9 kg/m^2^, and a mean excess weight of 173.63 ± 31.13%. Anthropometric assessment included the waist-to-hip ratio (WHR), with a mean value of 0.934 ± 0.07. Demographic and auxological characteristics are summarized in Table 1, while biochemical parameters are reported in Table 2.

Assessment of glucose metabolism showed that 22 children (66.7%) exhibited insulin resistance, defined by a HOMA-IR index > 2.5 and a pathological insulin response (IRI) curve. In this subgroup, serum glucose and insulin concentrations at different time points, together with HOMA-IR values, were analyzed to characterize glucose metabolism and explore their relationships with serum osteocalcin concentrations (Table 3 and Table 4). Despite the presence of insulin resistance, all patients maintained normal glucose tolerance and did not meet the diagnostic criteria for type 2 diabetes mellitus. Mean fasting plasma glucose was 88.10 ± 10.83 mg/dL and mean fasting insulin concentration was 24.91 ± 12.99.

**Figure 1:**
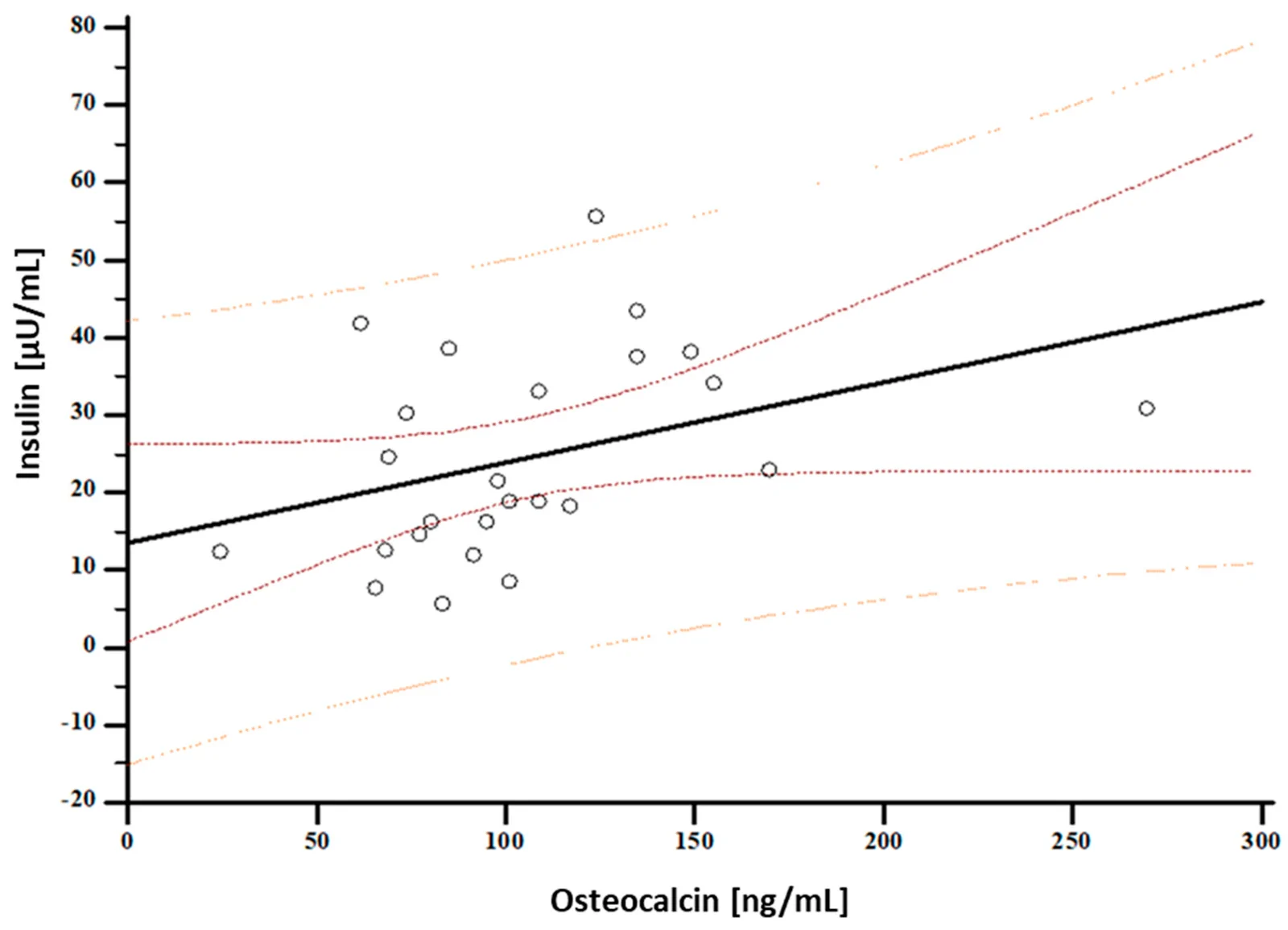
Correlation between osteocalcin levels and serum insulin concentrations.

**Figure 2:**
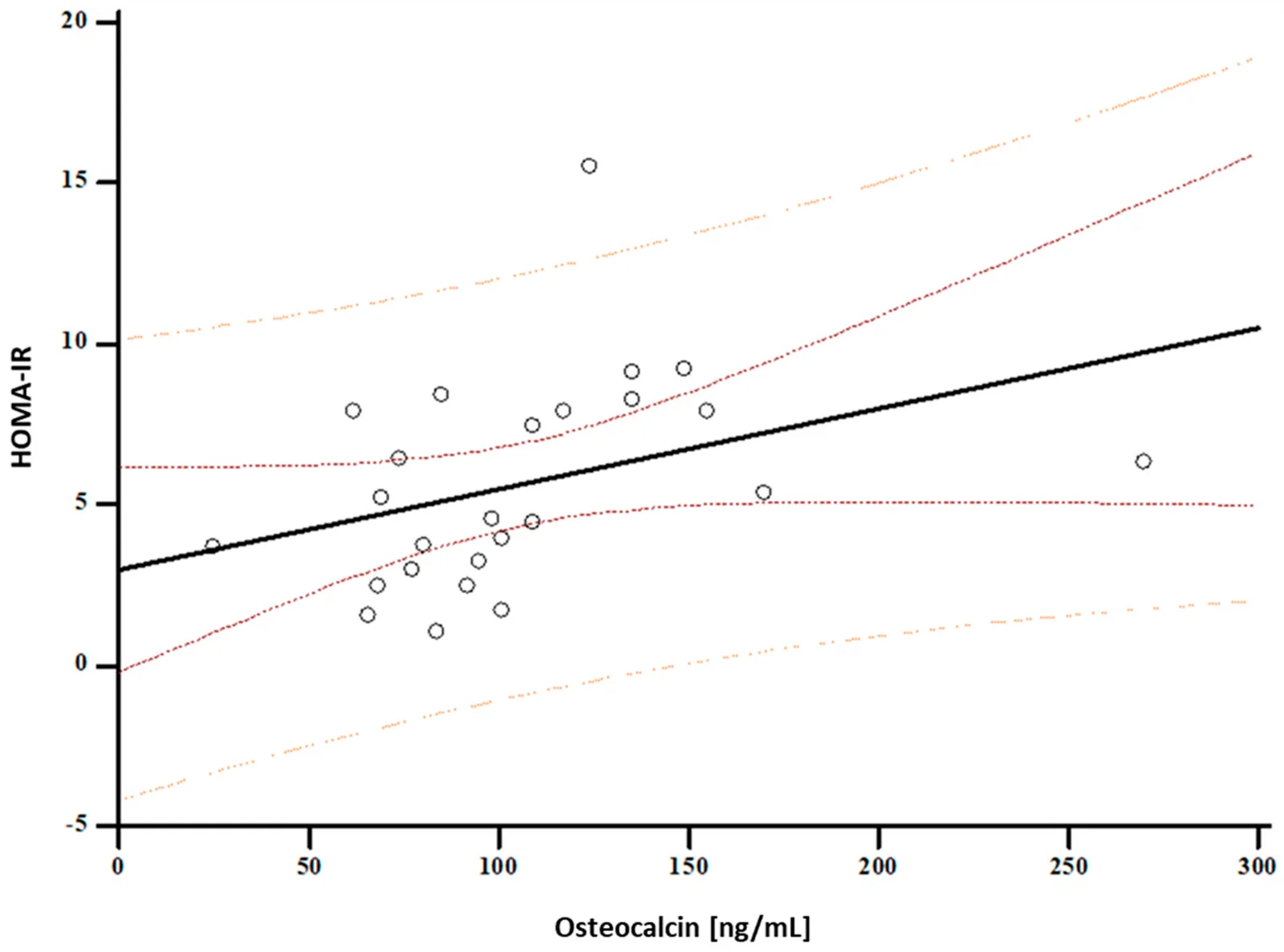
Correlation between osteocalcin levels and HOMA IR index.

Mean serum OC concentration was 112.1 ± 56.1 ng/mL and was significantly higher in obese children with insulin resistance. Exploratory correlation analyses, without formal adjustment for multiple comparisons, were subsequently performed to investigate potential associations between serum osteocalcin concentrations and the clinical, auxological, and biochemical characteristics of the study population. Therefore, *p*-values should be interpreted as exploratory. Non-parametric analysis showed a statistically significant positive correlation between serum osteocalcin levels and fasting insulin (*p* = 0.023) and between OC levels and the HOMA-IR index (*p* = 0.013) (Figure 1 and Figure 2).

No significant correlations were observed between serum OC levels and the following parameters: age (*p* = 0.774), bone age (*p* = 0.841), height (*p* = 0.904), BMI (*p* = 0.302), glucose (*p* = 0.155), GOT (*p* = 0.639), GPT (*p* = 0.844), total cholesterol (*p* = 0.105), HDL cholesterol (*p* = 0.986), LDL cholesterol (*p* = 0.083), triglycerides (*p* = 0.323), TSH (*p* = 0.671), 25-OH vitamin D (*p* = 0.985), CTx (*p* = 0.060), or PTH (*p* = 0.514). Open circles represent observations, and the solid black line represents the fitted linear regression line. The dashed lines indicate the associated statistical intervals.

## 4. Discussion

Considering the proposed involvement of osteocalcin (OC) in the regulation of glucose metabolism and insulin sensitivity, this exploratory study examined the relationship between circulating OC levels and early metabolic alterations in children with obesity and preserved glucose tolerance. In our cohort, higher OC concentrations were positively correlated with both the HOMA-IR index and fasting insulin levels. These observations may suggest, in pediatric obesity, a potential adaptive response to insulin resistance during the early stages of metabolic dysfunction [8,32]. Previous studies have explored the relationship between OC and glucose metabolism, suggesting a role for OC in the regulation of insulin sensitivity. However, the available evidence remains heterogeneous.

In adults with T2DM, lower OC levels are typically observed. Indeed, Lei et al., 2022 [33] reported an inverse correlation between serum OC and HOMA-IR (r = −0.153, *p* < 0.05) in adult Chinese patients with T2DM. Higher OC levels were associated with improved glycemic control, enhanced early-phase insulin secretion, and appropriate suppression of postprandial glucagon, supporting a potential role of OC in glucose homeostasis. Similarly, a cross-sectional retrospective study of 667 male patients with T2DM found negative associations between OC, glucose metabolism, and IR, though only when assessed via the triglyceride-glucose (TyG) index and not HOMA-IR [34]. Overall, these observations suggest that the association between osteocalcin and insulin resistance may differ between adults with established T2DM and children with obesity in the early stages of metabolic dysfunction. Further studies are needed to clarify the mechanisms underlying this relationship. However, the molecular pathways underlying this association have not yet been fully elucidated in pediatric populations.

Previous pediatric studies have reported conflicting results regarding OC and IR. Reinehr & Roth, 2010 [35] reported lower osteocalcin (OC) levels in obese children compared with normal-weight controls. Furthermore, total OC was negatively associated with HOMA-IR in both cross-sectional and longitudinal analyses, suggesting an inverse relationship between OC and insulin resistance. Conversely, Nassar et al., 2024 [36] observed no statistically significant correlations between OC and HOMA-IR, fasting insulin, or glucose in obese children, although trends were in the expected negative direction. Additional evidence from larger pediatric cohorts has demonstrated inverse associations between OC and metabolic risk factors such as triglycerides and waist circumference. However, these results have not been consistently replicated across different cohorts and analytical models [37]. Studies evaluating ucOC have similarly reported no significant associations with HOMA-IR after adjustment for adiposity, underscoring the inconsistency of current evidence in pediatric populations [38].

Furthermore, Redondo et al. 2017 [39] reported a negative correlation between OC and HbA1c shortly after T1DM onset, suggesting that elevated OC during early stages of the disease may transiently enhance insulin secretion. These discrepancies may reflect complex interactions among adiposity, cytokine profiles, developmental stage, and methodological differences, including the assessment of total versus undercarboxylated osteocalcin, sample size, and pubertal status.

Bilotta et al. 2018 described a reciprocal interaction between osteoblasts and pancreatic β-cells, in which insulin signaling in osteoblasts promotes osteocalcin (OC) production, while OC has been implicated in the regulation of insulin secretion [40]. This evidence supports the biological relevance of investigating the relationship between OC and glucose metabolism; however, the potential mechanisms underlying this association remain incompletely understood.

In line with this, the elevated OC levels observed in our cohort of children with obesity and insulin resistance, despite preserved glucose tolerance, may reflect a context-dependent metabolic adaptation associated with the early stages of metabolic dysfunction. However, the relationship between OC and β-cell function remains unclear. Further evidence regarding the association between OC and glucose metabolism comes from a separate cross-sectional study conducted at our center in children and adolescents with newly diagnosed T1DM. In that study, OC levels were positively correlated with stimulated *C*-peptide concentrations, a marker of residual β-cell function (r = 0.538; *p* = 0.026) [41]. Given the role of *C*-peptide as an indicator of endogenous insulin secretion, this finding suggests a possible association between OC levels and residual β-cell function in the early stages of T1DM, although no causal relationship can be inferred. Furthermore, lower OC levels have been reported in children with diabetic ketoacidosis, a condition associated with severe insulin deficiency; however, this association was not formally investigated in our cohort [41]. In the present study, the association between OC levels and insulin resistance in children with obesity highlights the need for further investigation. The clinical significance and underlying mechanisms of this relationship remain uncertain. Several limitations of the present study should also be acknowledged. The single-center design, combined with a relatively small sample size, may limit the external validity of our results. Other unmeasured factors, such as pubertal stage and adiposity distribution, may have acted as confounders in the observed associations between OC levels and insulin resistance. However, further longitudinal studies in larger, well-characterized pediatric cohorts are needed to clarify the temporal relationship between OC levels and β-cell function, and to evaluate whether OC may serve as an early biomarker of metabolic dysregulation.

## 5. Conclusions

The relationship between osteocalcin (OC) and insulin resistance in pediatric obesity appears complex and may vary according to metabolic status and developmental stage. In our cohort, higher serum OC levels were associated with insulin resistance and hyperinsulinemia despite preserved glucose tolerance. However, the clinical significance and underlying mechanisms of this association remain unclear. Larger longitudinal studies, including comprehensive assessments of circulating OC forms and β-cell function, are needed to clarify the temporal relationship between OC levels and metabolic dysfunction. Given the observational design of the present study, no causal relationship or compensatory role of OC in β-cell function can be inferred.

## 6. Limitations

Several limitations should be considered when interpreting our study. The retrospective, observational, single-center design precludes any inference of causality and may have introduced selection and information bias. In addition, the relatively small sample size limits statistical power and did not allow for more extensive subgroup analyses. Given the exploratory nature of the analyses and the relatively small sample size, stratified and robust multivariable analyses could not be performed. Therefore, the observed associations should be interpreted with caution and further investigated in larger powered cohorts.

The cross-sectional nature of the study also prevents assessment of the temporal relationship between circulating osteocalcin levels and insulin resistance. Although relevant clinical and biochemical variables were considered, residual confounding cannot be excluded, particularly regarding factors that may influence both bone and metabolic health, such as pubertal status, physical activity, dietary habits, body composition, and psychological factors, including stress and anxiety potentially associated with hormonal fluctuations during growth and puberty. In addition, a single HOMA-IR cut-off value (>2.5) was applied across the entire cohort. While this threshold has been previously used in pediatric populations, physiological variations related to age, sex, and pubertal development may influence HOMA-IR values. Finally, our study population consisted exclusively of pediatric patients with obesity and preserved glucose tolerance recruited from a specialized pediatric center; therefore, the results may not be generalizable to children with normal weight or to those with impaired glucose tolerance or type 2 diabetes. Nevertheless, as an exploratory study, it provides preliminary evidence and may serve as a basis for future research aimed at clarifying the role of osteocalcin in pediatric metabolic dysfunction.

## Figures and Tables

**Table 1:** Demographic and auxological characteristics of the study populations.

Demographic and Auxological Parameters	
Age (years)	10.4 ± 2.9
Bone age (years)	10.6 ± 3
Sex [male/female]	13/20
Weight (kg)	67.3 ± 20.7
Height (cm)	143.4 ± 14.8
BMI (kg/m^2^)	32 ± 5.9
Waist-to-hip ratio	0.9 ± 0.1

Data are reported as mean ± SD.

**Table 2:** Metabolic profile of the study populations.

Biochemical Assessments	
**Glucose and Insulin Resistance**	
Glucose [mg/dL]	88.1 ± 10.8
Insulin [μU/mL]	24.9 ± 13
HOMA-IR	5.6 ± 2.9
**Liver Enzymes**	
AST (GOT) [U/L]	20.2 ± 5.1
ALT (GPT) [U/L]	19.9 ± 7.1
**Lipid Profile**	
T-chol [mg/dL]	168.3 ± 23.9
HDL-chol [mg/dL]	47.6 ± 11.5
LDL-chol [mg/dL]	97.3 ± 23.3
TG [mg/dL]	94.7 ± 42
**Thyroid Function**	
TSH [mU/L]	2.3 ± 1.1
FT3 [pmol/L]	5.1 ± 3.1
FT4 [pmol/L]	16.1 ± 3.4
**Bone and Mineral Metabolism**	
Osteocalcin [ng/mL]	112.1 ± 56.1
CTx [ng/mL]	1.8 ± 0.7
IGF-1 [ng/mL]	233.3 ± 136.1
PTH [pg/mL]	31.2 ± 14.2
ALP [U/L]	256.3 ± 89.2
25-OH-D [ng/mL]	20.2 ±5.8
Calcium [mg/dL]	9.9 ±0.4
Phosphate [mg/dL]	4.9 ±0.7

ALP, alkaline phosphatase; CTx, *C*-terminal telopeptide of type I collagen; FT3, free triiodothyronine; FT4, free thyroxine; AST/GOT, glutamic-oxaloacetic transaminase; ALT/GPT, glutamic-pyruvic transaminase; HDL, high-density lipoprotein; HOMA-IR, Homeostatic Model Assessment of Insulin Resistance; IGF-1, insulin-like growth factor 1; LDL, low-density lipoprotein; 25-OH-D, 25-hydroxyvitamin D; PTH, parathyroid hormone; T-chol, total cholesterol; TG, triglycerides; TSH, thyroid-stimulating hormone. Data are reported as mean ± SD.

**Table 3:** HOMA-IR index and serum insulin concentrations at different time points.

HOMA-IR Index	Insulin [μU/mL] T0	Insulin [µU/mL] T30	Insulin [µU/mL] T60	Insulin [µU/mL] T90	Insulin [µU/mL] T120
6.41	29.5	254	166	185	144
7.93	41.7	308	51.2	146	124
15.51	55.6	286	163	372	389
3.22	16.1	78.4	204	133	61
3.71	12.3	114	107	175	136
8.26	37.6	247	181	53.2	83.4
2.99	14.6	78.6	61.8	61.8	65.4
3.95	25.3	168	262	81.4	203
4.43	18.9	333	262	128	205
9.11	43.4	159	155	292	268
5.2	24.5	67.4	186	74	82
8.39	38.6	295	248	249	439
9.24	38.2	252	207	167	181
7.91	34.1	12	218	249	185
6.33	30.5	349	403	81.4	203
7.91	18.2	155	44.7	77.9	64.1
3.72	16.2	184	233	149	120
4.05	16.9	99	115	68	77.6
6.78	28.6	110	139	167	169
9.5	47.5	218	148	262	133
4.54	21.4	132	79.3	85.8	173
7.44	33.1	159	197	100	199

HOMA-IR index and serum insulin concentrations at baseline (T0) and 30, 60, 90, and 120 min (T30, T60, T90, and T120, respectively) following an oral glucose tolerance test (OGTT; glucose dose: 1.75 g/kg body weight, up to a maximum of 75 g) in patients with insulin resistance (*n* = 22). Data are expressed as mean ± standard deviation (SD). HOMA-IR, homeostatic model assessment of insulin resistance; OGTT, oral glucose tolerance test.

**Table 4:** Serum glucose concentrations at different time points.

Glucose [mg/dL] T0′	Glucose [mg/dL] T30′	Glucose [mg/dL] T60′	Glucose [mg/dL] T90′	Glucose [mg/dL] T120′
113	183	154	155	125
100	145	84	90	101
103	140	119	110	115
113	168	133	149	138
114	151	157	142	113
122	149	152	145	157
105	132	125	104	120
92	123	97	111	108
101	146	154	132	126
86	99	123	112	91
89	154	157	102	110
83	127	130	106	120
88	124	102	117	130
98	163	132	123	126
88	140	127	96	119
87	183	175	94	82
93	142	145	116	105
97	183	140	91	121
96	149	150	140	110
97	127	110	129	103
99	151	138	133	141
91	113	134	106	124

Blood glucose concentrations at baseline (T0) and 30, 60, 90, and 120 min (T30, T60, T90, and T120, respectively) following an oral glucose tolerance test (OGTT; glucose dose: 1.75 g/kg body weight, up to a maximum of 75 g) in patients with insulin resistance (*n* = 22). Data are expressed as mean ± standard deviation (SD). OGTT, oral glucose tolerance test.

## Data Availability

The data supporting the findings of this study are not publicly available due to privacy and ethical restrictions, as they consist of clinical data derived from patient medical records. The data are available from the corresponding author upon reasonable request and subject to applicable privacy and ethical restrictions.
